# Malarial retinopathy in adult: a case report

**DOI:** 10.11604/pamj.2017.27.224.11026

**Published:** 2017-07-25

**Authors:** Chantal Ngoune Nanfack, Yannick Bilong, Giles Kagmeni, Ngoulou Ngoulou Nathan, Lucienne Assumpta Bella

**Affiliations:** 1 Ophthalmology Unit, Pediatric and Gynéco-Obstetric Hospital, Yaoundé, Cameroon; 2 Department of Ophthalmology, Faculty of Medicine and Biomedical Sciences, University of Yaoundé I, Yaoundé, Cameroon

**Keywords:** Cerebral malaria, retinopathy, visual acuity, retinal hemorrhages

## Abstract

Although widely reported in children, the malarial retinopathy has rarely been described in adults. We observed a case in the department of ophthalmology at the pediatric and gynecology-obstetrics Yaoundé Hospital. The diagnosis revealing a decrease in visual acuity was confirmed by thorough ophthalmological and biological assessments. The basic treatment by **quinine therapy** was conclusive. The authors point out the need to consider this diagnosis in case of any decrease in visual acuity in febrile context for any adult living or recently having stayed in endemic areas.

## Introduction

Cerebral malaria is **a neurological complication of malaria** caused by plasmodium falciparum. Ophthalmological lesions have been described in this condition and some retinal **lesions** are specific to it [1]. They give rise to a disease entity: the malarial retinopathy. It was much described in children, often in a comatose context, and sometimes with fatal outcome [2]. On the contrary, it has been little reported in adults; so there is a need to enrich the literature with well documented observations. The aim of this work was to share a case of malarial retinopathy observed and documented, on an adult at the pediatric and gynecology-obstetrics Yaoundé in Cameroon, a country in sub-Saharan Africa.

## Patient and observation

**Figure 1:**
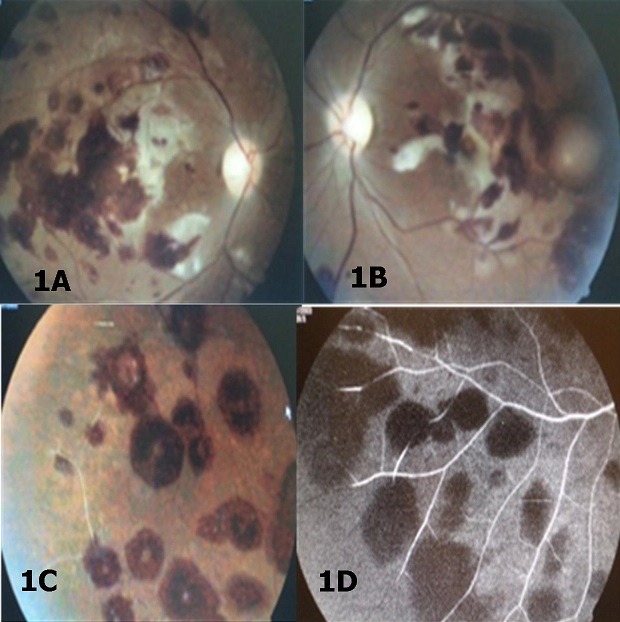
(A,B) retinal hemorrhage and retinal bleaching zones, C) discoloration of retinal vessels; D) retinal ischemia

**Figure 2:**
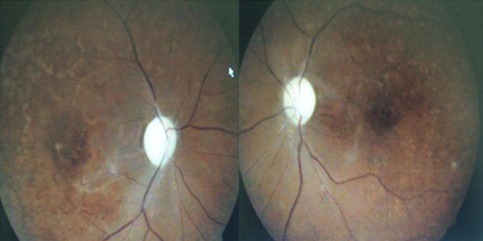
Regression of retinal signs left and right eye

A 29 year old male was consulted for a sudden drop in visual acuity and more severe at right eye, which occurred in febrile context. For the clinical examination: the patient was prostrate, visual acuity consisted of the patient being able to see a waving hand from his right eye, and a tenth of it from his left eye, with no improvement upon using both eyes. A bilateral reactive mydriasis was also present. Intraocular pressure measured 11 mmHg and 13mmhg on the right eye and left eye respectively. The eyes backgrounds revealed rounded retinal hemorrhages, some with clear centers scattered throughout the retina, more pronounced in the right eye; bleaching areas of the retina in the macular area more pronounced in the right eye (Figure 1, A,B). And discoloration of peripheral vessels on the inferior temporal in the right eye (Figure 1, C). The fluorescein angiography revealed: intra-retinal hemorrhages, retinal ischemia on the inferior temporal of the right eye (Figure 1 D). Thick smear was positive with two thousand trophozoides by field. The blood count was in favor of a microcytic hypochromic anemia with a hemoglobin rate of 11g / l. We noted a lack of immature cells in the blood smear. The support consisted of patient hospitalization and administration of quinine at a rate of 8 mg / kg diluted in 250cc serum glucose every 8 hours for 5 days. The evolution under treatment at Day 60 was marked by an improvement in visual acuity to a tenth in the right eye and three tenths in the left eye, in addition to a disappearance of retinal signs (Figure 2).

## Discussion

This work reports a case of malarial retinopathy that has been widely documented and treated successfully with optimal intravenous quinine therapy on an adult. Although this entity has been widely described in children, we were able to find only few cases reported for adults. So, there is a real interest in sharing the different cases observed for the purpose of increasing the general awareness of this entity whose diagnosis may be difficult. The malaria retinopathy was actually described for the first time by Lemallen et al in 1993 [3]. It might be caused by the occlusion of the secondary retinal microcirculation and sequestration of parasitized red blood cells [4]. Indeed, plasmodia have been found in choroidal vessels of enucleated globes of patients who died of malaria coma [5]. The types of retinal abnormalities found in our patient: whitening of the retina, discoloration of the vessels, and retinal hemorrhages have been described in the literature during cerebral malaria, the whitening of the retina and the bleaching vessel being specific to malaria retinopathy [2]. To this end, a study in Zaire on children under 6years of age with cerebral malaria, found retinopathy in 31% of cases [6]. In the same token, Nicholas et al found on a 2 year old patient admitted in a coma for cerebral malaria, a discoloration of the retinal vessels and bleaching areas of the retina [7]. On the other hand, Ebana et al detected the presence of macular and macular perished of rounded hemorrhages on a series of three patients [8].These rounded hemorrhages with a clear center are also described in hematological malignancies. However, the presence of specific signs of malarial retinopathy and the absence of immature cells in blood smear in our patient, allowed us to reject this hypothesis. During the malaria retinopathy, the impact on the visual acuity is observed when the lesions are located in the macular area; that was the case for our patient. Ebana et al also found some evidence in a decrease of visual acuity and metamorphopsia [8].

According to some authors, the severity of malaria retinopathy would increase with the severity of malaria [**3**] indeed, prospective studies conducted in children with cerebral malaria in Malawi and Kenya respectively, found that the severity of retinal signs was associated with fatal outcome and depth of coma in survivors [9]. Our patient showed a fairly good condition with very significant retinal signs. This might be explained by the fact that he is an adult who lives in a malaria endemic area, therefore, who has developed pre-immunity, whereas most studies have been conducted on children who are new subjects. This gravity is even more important in the presence of papilledema [10]. For instance, a study in Malawi found a mortality rate of 18% in patients with malarial retinopathy and without papilledema, as compared to 44% among those with malarial retinopathy and papilledema [10]. So, the absence of papilledema in our patient would justify his fairly good condition despite the severity of retinal signs. The malaria retinopathy can be resolved without persisting retinal lesions or symptoms [3]. A study on the visual impact does not find any detectable effect of malarial retinopathy on visual acuity in the first months out of the coma [8]. Ebana et al found an improvement in visual acuity, but with persistence metamorphopsia [8].

## Conclusion

Although primarily encountered during cerebral malaria in children, the malarial retinopathy also occurs in adults. The whitening of the retina and bleaching vessels are specific signs to the malarial retinopathy. When the lesions are located in the macular area, it causes an impact on the visual acuity.

## Competing interests

The authors declare no competing interests.
